# 1,25-dihydroxyvitamin D deficiency is independently associated with cardiac valve calcification in patients with chronic kidney disease

**DOI:** 10.1038/s41598-022-04981-x

**Published:** 2022-01-18

**Authors:** Il Young Kim, Byung Min Ye, Min Jeong Kim, Seo Rin Kim, Dong Won Lee, Hyo Jin Kim, Harin Rhee, Sang Heon Song, Eun Young Seong, Soo Bong Lee

**Affiliations:** 1 Department of Internal Medicine, Pusan National University School of Medicine, Yangsan, South Korea; 2 Research Institute for Convergence of Biomedical Science and Technology, Pusan National University Yangsan Hospital, Yangsan, South Korea; 3 Medical Research Institute, Pusan National University Hospital, Busan, South Korea

**Keywords:** Chronic kidney disease, Risk factors

## Abstract

Cardiac valve calcification is highly prevalent in patients with chronic kidney disease (CKD). Low vitamin D levels are associated with vascular calcification in CKD. However, the association between vitamin D levels and cardiac valve calcification is unknown. A total of 513 patients with pre-dialysis CKD were included in this cross-sectional study. Aortic valve calcification (AVC) and mitral valve calcification (MVC) were assessed using two-dimensional echocardiography. The associations between AVC and MVC and baseline variables were investigated using logistic regression analyses. In multivariable analysis, serum 1,25(OH)_2_D level was independently associated with AVC (odds ratio [OR], 0.87; P < 0.001) and MVC (OR, 0.92; P < 0.001). Additionally, age, diabetes, coronary heart disease, calcium × phosphate product, and intact parathyroid hormone levels were independently associated with AVC and MVC. Systolic blood pressure was independently associated with AVC. A receiver-operating characteristic (ROC) curve analysis showed that the best cutoff values of serum 1,25(OH)_2_D levels for predicting AVC and MVC were ≤ 12.5 and ≤ 11.9 pg/dl, respectively. Serum 1,25(OH)_2_D deficiency is independently associated with AVC and MVC in patients with CKD, suggesting that serum 1,25(OH)_2_D level may be a potential biomarker of AVC and MVC in these patients.

## Introduction

Cardiovascular disease (CVD) is highly prevalent and the most common cause of death in patients with chronic kidney disease (CKD)^1^. Cardiac valve calcification is a common complication of CVD and is associated with an increased risk of CVD and all-cause mortality in patients with CKD^2–5^. The Kidney Disease: Improving Global Outcome (KDIGO) addressed the clinical relevance of cardiac valve calcification in CKD and suggested that cardiac valve calcification should be included in the risk stratification of CVD in patients with CKD^6^.

Multiple contributors, including traditional factors (age, hypertension, diabetes, and dyslipidemia) as well as non-traditional factors (hyperphosphatemia, calcium phosphate product, and parathyroid function), have been suggested to be involved in cardiac valve calcification in patients with CKD^6^. Vitamin D plays a central role not only in bone metabolism but also in the vasculature and may be involved in the process of vascular calcification^7^. Indeed, low levels of 25-hydroxyvitamin D [25(OH)D] or 1,25-dihydroxyvitamin D [1,25(OH)_2_D] have been reported to be associated with coronary artery and cardiac valve calcification in patients with risk factors for CVD and in the general population^8–10^. Vitamin D metabolism is ubiquitously altered in patients with CKD, and both 1,25(OH)_2_D and 25(OH)D levels are insufficient in the majority of patients with CKD^11^. Although an independent association between low levels of 25(OH)D and coronary artery calcification has been previously reported in patients with CKD^12,13^, whether vitamin D deficiency is associated with cardiac valve calcification in patients with CKD is still unknown.

In this study, we hypothesized that vitamin D deficiency would be independently associated with cardiac valve calcification and investigated the association between serum 1,25(OH)_2_D levels and aortic valve calcification (AVC) and mitral valve calcification (MVC) in patients with CKD.

## Methods

### Study population

From 2010 to 2019, the data of 513 patients who visited the nephrology clinic at Pusan National University Yangsan Hospital were retrospectively investigated. The estimated glomerular filtration rate (eGFR) was determined using the Modification of Diet in Renal Disease equation: 186 × serum creatinine levels^−1.154^ × patient age^−0.203^ × 0.742 (if female) or × 1.21 (if African-American)^14^. All enrolled patients were adults (≥ 18 years old) with CKD (eGFR < 60 ml/min/1.73 m^2^) and not on dialysis. To exclude patients with acute kidney injury and to include only patients with CKD, we included only patients whose previous serum creatinine levels were known from medical records or who had been followed for at least three months. We also excluded patients who had taken vitamin D supplements, calcimimetics, or phosphate binders, which could affect the endogenous 1,25(OH)_2_D metabolism. The study protocol was approved by the Institutional Review Board of Pusan National University Yangsan Hospital (IRB No. 05-2021-119). All research and data collection processes were conducted in accordance with the Declaration of Helsinki and current ethical guidelines. The Institutional Review Board of Pusan National University Yangsan Hospital waived the need for informed consent due to the retrospective nature of the analysis, which only used the information available from anonymized medical charts and records.

### Data collection

Demographic and clinical data regarding age, sex, current smoking status, history of diabetes, history of CVD (coronary heart disease, cerebrovascular disease, and peripheral vascular disease), history of medication use (angiotensin-converting enzyme inhibitors [ACEI], angiotensin receptor blockers [ARB], calcium channel blockers, beta-blockers, thiazide/loop diuretics, anti-platelet agents, and statins), body mass index (BMI), and blood pressure were obtained by reviewing the medical records, based on our previous report^15^. Diabetes was defined as the use of diabetes medication, fasting plasma glucose concentration ≥ 126 mg/dl, or A1c hemoglobin levels ≥ 6.5%. Blood pressure was measured from each patient’s upper right arm in a sedentary position using an automated sphygmomanometer after a 5-min rest. BMI was calculated by measuring each patient’s weight and height and was expressed as kg/m^2^. All serum parameters, including albumin, uric acid, calcium, phosphate, total cholesterol, hemoglobin, C-reactive protein (CRP), intact parathyroid hormone (PTH), and 1,25(OH)_2_D levels, were measured concomitantly. Serum 1,25(OH)2D levels were measured by a radioimmunoassay (DIAsource ImmunoAssays, Louvain-la-Neuve, Belgium) (reference range 19.6–54.3 pg/ml in healthy adults). The amount of urinary albumin was measured by calculating the urinary albumin-to-creatinine ratio (mg/g Cr).

### Echocardiography

Two-dimensional echocardiography was performed using an IE33 echo system (Philips, Amsterdam, Netherlands), based on our previous report^16^. All echocardiographic data were acquired according to the guidelines of the American Society of Echocardiography^17^ and were analyzed by an experienced cardiologist who was blinded to the clinical details. The presence of AVC and MVC was determined by visual inspection. Cardiac valve calcification was defined as bright echoes of more than 1 mm in length on one or more cusps of the aortic or mitral valves.

### Statistical analysis

Statistical analysis was performed based on our previous report^15^. Continuous variables are expressed as means ± standard deviations, while categorical variables are presented as percentages. Comparisons between the three CKD stage groups or three 1,25(OH)2D tertile groups were performed with a one-way analysis of variance for continuous variables and the chi-square test for categorical variables. Univariate and multivariable logistic regression analyses were used to determine the factors for predicting the presence of AVC, MVC, and at least one valve calcification (AVC or MVC). Significant variables were identified by univariate analysis, and the clinically important variables were selected for multivariable analysis. A receiver-operating characteristic (ROC) curve analysis was performed to assess the area under the curve (AUC) and Youden index was used to determine the best cutoff value of serum 1,25(OH)2D levels for predicting the presence of AVC, MVC, or at least one valve calcification. Statistical significance was set at P < 0.05. All analyses were performed using the SPSS version 26.0 (SPSS, Inc., Chicago, IL, USA) and MedCalc Statistical Software version 19.4.1 (MedCalc Software, Ostend, Belgium).

## Results

### Study population

Baseline characteristics of the study population according to CKD stage were summarized in Supplementary Table S1. Of the 513 patients included in the study, 271 had CKD stage 3; 181, CKD stage 4; and 61, CKD stage 5. The mean eGFRs was 42.2 ± 8.2, 22.1 ± 4.6, and 10.2 ± 4.5 ml/min/1.73 m^2^ in the CKD stages 3, 4, and 5 groups, respectively (P < 0.001). Regarding the biochemical parameters of CKD-mineral and bone disorder (MBD), patients with higher CKD stages were likely to have elevated serum levels of phosphate (P < 0.001), calcium × phosphate (Ca × P) product (P < 0.001), and intact PTH (P < 0.001), whereas they were likely to have decreased serum levels of calcium (P < 0.001). The levels of 1,25(OH)_2_D were significantly different between the three CKD stage groups (CKD stages 3 vs. 4 vs. 5: 22.0 ± 10.7 pg/dl vs. 15.1 ± 9.1 pg/dl vs. 8.3 ± 3.9 pg/dl, P < 0.001). Patients with higher CKD stages were likely to have a higher prevalence of AVC (CKD stages 3 vs. 4 vs. 5: 8.5% vs. 25.4% vs. 49.2%, P < 0.001), MVC (CKD stages 3 vs. 4 vs. 5: 7.7% vs. 19.9% vs. 39.3%, P < 0.001), and at least one valve calcification (CKD stages 3 vs. 4 vs. 5: 12.9% vs. 32.0% vs. 55.7%, P < 0.001) than that seen in patients with lower CKD stages.

Table 1 shows the baseline characteristics of three 1,25(OH)2D tertile groups. There were significant differences between the three groups in age (P = 0.001); prevalence of diabetes (P = 0.007) and coronary heart disease (P = 0.014); systolic (P < 0.001) and diastolic (P = 0.006) blood pressure; levels of urinary albumin (P = 0.001) and serum phosphate (P < 0.001), Ca × P product (P < 0.001), hemoglobin (P < 0.001), CRP (P < 0.001), intact PTH (P < 0.001), eGFR (P = 0.001), and serum albumin level (P < 0.001). There were also significant differences between the three groups in prevalence of AVC (P < 0.001), MVC (P < 0.001), and at least one valve calcification (P < 0.001).

**Table 1:** Baseline characteristics of the study population according to tertiles of serum 1,25(OH)_2_D levels (n = 513).

	Tertile 1 (< 11.5 pg/dl)	Tertile 2 (11.5–21.4 pg/dl)	Tertile 3 (> 21.4 pg/dl)	P
Age (years)	61.9 ± 10.5	61.2 ± 9.7	58.1 ± 10.0	0.001
Sex, male [n (%)]	86 (51.5%)	88 (50.6%)	89 (51.7%)	0.974
Current smoking [n (%)]	25 (15.0%)	25 (14.4%)	35 (20.3%)	0.260
Diabetes [n (%)]	88 (52.7%)	103 (59.2%)	73 (42.4%)	0.007
**Cardiovascular disease [n (%)]**				
Coronary heart disease^a^	47 (28.1%)	38 (21.8%)	26 (15.1%)	0.014
Cerebrovascular disease^b^	17 (10.2%)	12 (6.9%)	25 (14.5%)	0.068
Peripheral vascular disease	15 (9.0%)	16 (9.2%)	7 (4.1%)	0.122
**Medication [n (%)]**				
ACEI or ARB	129 (77.2%)	133 (76.4%)	129 (78.0%)	0.886
Calcium channel blockers	111 (66.5%)	103 (59.2%)	98 (57.0%)	0.174
Beta-blockers	68 (40.7%)	67 (38.5%)	60 (34.9%)	0.535
Diuretics (thiazide)	41 (24.6%)	53 (30.5%)	59 (34.3%)	0.142
Diuretics (loop)	81 (48.5%)	73 (42.0%)	66 (38.4%)	0.162
Anti-platelet agents	57 (34.1%)	61 (35.1%)	40 (23.3%)	0.031
Statins	66 (39.5%)	60 (34.5%)	54 (31.4%)	0.587
Body mass index (kg/m^2^)	23.8 ± 2.3	23.5 ± 2.5	23.7 ± 2.4	0.603
Systolic blood pressure (mmHg)	140.7 ± 19.3	134.2 ± 17.9	128.6 ± 19.8	< 0.001
Diastolic blood pressure (mmHg)	81.8 ± 15.1	78.8 ± 12.7	77.0 ± 13.5	0.006
eGFR (ml/min/1.73 m^2^)	23.1 ± 12.2	33.8 ± 13.8	36.7 ± 11.5	< 0.001
Urinary albumin (mg/g Cr)	1473.5 ± 1322.6	1230.7 ± 1113.0	1010.4 ± 975.4	0.001
Albumin (g/dl)	4.0 ± 0.4	4.1 ± 0.4	4.2 ± 0.3	< 0.001
Uric acid (mg/dl)	7.7 ± 2.7	7.1 ± 2.9	7.4 ± 2.7	0.216
Calcium (mg/dl)	9.2 ± 0.6	9.2 ± 0.7	9.3 ± 0.4	0.820
Phosphate (mg/dl)	4.6 ± 1.0	4.1 ± 0.9	3.6 ± 0.7	< 0.001
Ca × P product (mg^2^/dl^2^)	42.6 ± 10.0	37.5 ± 8.5	33.7 ± 6.5	< 0.001
Total cholesterol (mg/dl)	215.1 ± 42.4	212.8 ± 44.3	205.7 ± 37.4	0.094
Hemoglobin (g/dl)	11.0 ± 1.8	11.9 ± 1.8	12.2 ± 1.8	< 0.001
CRP (mg/dl)	1.0 ± 1.1	0.7 ± 0.6	0.8 ± 0.8	< 0.001
Intact PTH (pg/ml)	124.1 ± 67.0	81.4 ± 56.6	66.3 ± 45.1	< 0.001
Aortic valve calcification [n (%)]	71 (42.5%)	23 (13.2%)	5 (2.9%)	< 0.001
Mitral valve calcification [n (%)]	54 (32.3%)	20 (11.5%)	7 (4.1%)	< 0.001
At least one valve calcification [n (%)]	86 (51.5%)	32 (18.4%)	9 (5.2%)	< 0.001

Data are presented as mean ± standard deviation or (n, %). ^a^Coronary heart disease is defined as a history of coronary artery bypass surgery or percutaneous transluminal coronary angioplasty. ^b^Cerebrovascular disease is defined as a history of stroke or transient ischemic attack.

*ACEI* angiotensin-converting enzyme inhibitor, *ARB* angiotensin receptor blockers, *Ca × P* product, calcium × phosphorus product, *CKD* chronic kidney disease, *CRP* C-reactive protein, *eGFR* estimated glomerular filtration rate, *PTH* parathyroid hormone, *1,25(OH)*_*2*_*D* 1,25-dihydroxyvitamin D.

### Association between serum 1,25(OH)_2_D levels and cardiac valve calcification

The association between the presence of AVC and baseline variables are shown in Table 2. A univariate logistic regression analysis revealed that age, diabetes, coronary heart disease, cerebrovascular disease, systolic and diastolic blood pressure, eGFR, and the levels of urinary albumin and serum albumin, Ca × P product, hemoglobin, CRP, intact PTH, and 1,25(OH)_2_D were significantly associated with AVC. In a multivariable logistic regression analysis, serum 1,25(OH)_2_D level (odds ratio [OR]: 0.87, 95% confidence interval [CI]: 0.82–0.91, P < 0.001) was independently associated with AVC. In addition, age (OR: 0.016, 95% CI 1.00–1.08, P = 0.016), diabetes (OR: 2.07, 95% CI 1.06–4.03, P = 0.033), coronary heart disease (OR: 2.71, 95% CI 1.18–6.24, P = 0.019), systolic blood pressure (OR: 1.29, 95% CI 1.03–1.62, P = 0.030), and serum Ca × P product (OR: 1.05, 95% CI 1.01–1.10, P = 0.001) and intact PTH levels (OR: 1.14, 95% CI 1.04–1.24, P < 0.001) were independently associated with AVC.

**Table 2:** Univariate and multivariable analyses for variables associated with aortic valve calcification in the study population (n = 513).

	Univariate		Multivariable	
	Odds ratio (95% CI)	P	Odds ratio (95% CI)	P
Age (1 year)	1.08 (1.05–1.10)	< 0.001	1.04 (1.00–1.08)	0.016
Sex, male	1.01 (0.65–1.57)	0.956	1.09 (0.58–2.03)	0.796
Current smoking	1.36 (0.78–2.37)	0.281		
Diabetes	2.18 (1.38–3.46)	0.001	2.07 (1.06–4.03)	0.033
**Cardiovascular disease**				
Coronary heart disease^a^	3.47 (2.16–5.59)	< 0.001	2.71 (1.18–6.24)	0.019
Cerebrovascular disease^b^	1.91 (1.02–3.58)	0.045	1.19 (0.41–3.44)	0.755
Peripheral vascular disease	1.55 (0.73–3.31)	0.258	1.91 (0.62–5.89)	0.263
**Medication**				
ACEI or ARB	1.51 (0.87–2.64)	0.147		
Calcium channel blockers	1.10 (0.70–1.73)	0.682		
Beta-blockers	1.32 (0.85–2.07)	0.217		
Diuretics (thiazide)	0.66 (0.40–1.10)	0.112		
Diuretics (loop)	1.12 (0.77–1.86)	0.423		
Anti-platelet agents	1.37 (0.86–2.17)	0.183		
Statins	1.33 (0.85–2.08)	0.218		
Body mass index (1 kg/m^2^)	1.07 (0.98–1.17)	0.152		
Systolic blood pressure (10 mmHg)	1.63 (1.41–1.89)	< 0.001	1.29 (1.03–1.62)	0.030
Diastolic blood pressure (10 mmHg)	1.46 (1.24–1.72)	< 0.001	1.03 (0.78–1.36)	0.818
eGFR (1 ml/min/1.73 m^2^)	0.93 (0.91–0.95)	< 0.001	1.01 (0.97–1.05)	0.722
Urinary albumin (100 mg/g Cr)	1.03 (1.01–1.05)	0.003	0.99 (0.96–1.02)	0.532
Albumin (1 ng/dl)	0.55 (0.31–0.96)	0.034	2.02 (0.75–5.45)	0.165
Uric acid (1 mg/dl)	1.06 (0.98–1.15)	0.125		
Ca × P product (1 mg^2^/dl^2^)	1.13 (1.10–1.16)	< 0.001	1.05 (1.01–1.10)	0.011
Total cholesterol (1 mg/dl)	1.00 (1.00–1.01)	0.513		
Hemoglobin (1 g/dl)	0.75 (0.66–0.85)	< 0.001	1.12 (0.91–1.37)	0.290
CRP (1 mg/dl)	1.85 (1.45–2.36)	< 0.001	1.33 (0.92–1.91)	0.125
Intact PTH (10 pg/ml)	1.21 (1.16–1.26)	< 0.001	1.14 (1.04–1.24)	0.004
1,25(OH)_2_D (1 pg/dl)	0.84 (0.81–0.88)	< 0.001	0.87 (0.82–0.91)	< 0.001

Data are presented as odds ratio and 95% confidence interval (CI). ^a^Coronary heart disease is defined as a history of coronary artery bypass surgery or percutaneous transluminal coronary angioplasty. ^b^Cerebrovascular disease is defined as a history of stroke or transient ischemic attack.

*ACEI* angiotensin-converting enzyme inhibitors, *ARB* angiotensin receptor blockers, *Ca × P* product, calcium × phosphorus product, *CKD* chronic kidney disease, *CRP* C-reactive protein, *eGFR* estimated glomerular filtration rate, *PTH* parathyroid hormone, *1,25(OH)*_*2*_*D* 1,25-dihydroxyvitamin D.

Table 3 shows the baseline variables associated with the presence of MVC. A univariate logistic regression analysis revealed that age, diabetes, coronary artery disease, cerebrovascular disease, systolic and diastolic blood pressure, eGFR, and the levels of urinary albumin and serum albumin, Ca × P product, hemoglobin, CRP, intact PTH, and 1,25(OH)_2_D were significantly associated with MVC. A multivariable logistic regression analysis revealed that 1,25(OH)_2_D level (OR, 0.92; 95% CI 0.88–0.97, P = 0.001) was independently associated with MVC. Furthermore, age (OR: 1.04, 95% CI 1.00–1.07, P = 0.030), diabetes (OR: 2.28, 95% CI 1.19–4.36, P = 0.013), coronary heart disease (OR: 2.77, 95% CI 1.27–6.05, P = 0.011), and serum Ca × P product (OR: 1.04, 95% CI 1.01–1.08, P = 0.025) and intact PTH levels (OR: 1.09, 95% CI 1.01–1.18, P = 0.024) were independently associated with MVC.

**Table 3:** Univariate and multivariable analyses for variables associated with mitral valve calcification in the study population (n = 513).

	Univariate		Multivariable	
	Odds ratio (95% CI)	P	Odds ratio (95% CI)	P
Age (1 year)	1.09 (1.06–1.12)	< 0.001	1.04 (1.00–1.07)	0.030
Sex, male	0.91 (0.57–1.47)	0.712	0.86 (0.48–1.57)	0.632
Current smoking	0.97 (0.50–1.82)	0.891		
Diabetes	2.58 (1.55–4.31)	< 0.001	2.28 (1.19–4.36)	0.013
**Cardiovascular disease**				
Coronary heart disease^a^	3.34 (2.01–5.53)	< 0.001	2.77 (1.27–6.05)	0.011
Cerebrovascular disease^b^	2.55 (1.35–4.84)	0.004	2.06 (0.78–5.44)	0.146
Peripheral vascular disease	1.74 (0.79–3.82)	0.170		
**Medication**				
ACEI or ARB	1.11 (0.63–1.96)	0.720		
Calcium channel blockers	1.19 (0.72–1.94)	0.498		
Beta-blockers	1.46 (0.90–2.35)	0.123		
Diuretics (thiazide)	0.68 (0.39–1.19)	0.174		
Diuretics (loop)	1.29 (0.80–2.07)	0.298		
Anti-platelet agents	1.07 (0.65–1.79)	0.783		
Statins	1.18 (0.72–1.92)	0.513		
Body mass index (1 kg/m^2^)	1.00 (0.91–1.10)	0.973		
Systolic blood pressure (10 mmHg)	1.43 (1.24–1.65)	< 0.001	1.09 (0.88–1.36)	0.431
Diastolic blood pressure (10 mmHg)	1.32 (1.11–1.57)	0.002	0.94 (0.73–1.22)	0.651
eGFR (1 ml/min/1.73 m^2^)	0.93 (0.91–0.95)	< 0.001	1.01 (0.97–1.05)	0.661
Urinary albumin (100 mg/g Cr)	1.03 (1.01–1.05)	0.001	0.99 (0.96–1.02)	0.666
Albumin (1 ng/dl)	0.45 (0.25–0.81)	0.008	1.36 (0.54–3.34)	0.510
Uric acid (1 mg/dl)	1.06 (0.98–1.16)	0.168		
Ca × P product (1 mg^2^/dl^2^)	1.12 (1.09–1.15)	< 0.001	1.04 (1.01–1.08)	0.025
Total cholesterol (1 mg/dl)	1.00 (0.99–1.01)	0.818		
Hemoglobin (1 g/dl)	0.68 (0.59–0.79)	< 0.001	0.91 (0.75–1.10)	0.319
CRP (1 mg/dl)	1.46 (1.15–1.85)	0.002	0.95 (0.67–1.36)	0.790
Intact PTH (10 pg/ml)	1.19 (1.14–1.24)	< 0.001	1.09 (1.01–1.18)	0.024
1,25(OH)_2_D (1 pg/dl)	0.89 (0.86–0.92)	< 0.001	0.92 (0.88–0.97)	0.001

Data are presented as odds ratio and 95% confidence interval (CI). ^a^Coronary heart disease is defined as a history of coronary artery bypass surgery or percutaneous transluminal coronary angioplasty. ^b^Cerebrovascular disease is defined as a history of stroke or transient ischemic attack.

*ACEI* angiotensin-converting enzyme inhibitors, *ARB* angiotensin receptor blockers, *Ca × P* product, calcium × phosphorus product, *CKD* chronic kidney disease, *CRP* C-reactive protein, *eGFR* estimated glomerular filtration rate, *PTH* parathyroid hormone, *1,25(OH)*_*2*_*D* 1,25-dihydroxyvitamin D.

Table 4 shows the baseline variables associated with the presence of at least one valve calcification (AVC or MVC). A univariate logistic regression analysis showed that age, diabetes, coronary heart disease, cerebrovascular disease, systolic and diastolic blood pressure, eGFR, and the levels of urinary albumin and serum albumin, Ca × P product, hemoglobin, CRP, intact PTH, and 1,25(OH)_2_D were associated with at least one valve calcification. Multivariable logistic regression analysis showed that serum 1,25(OH)_2_D level (OR: 0.88, 95% CI 0.84–0.92, P < 0.001) was an independent predictor of at least one valve calcification. Age (OR: 1.05, 95% CI 1.01–1.09, P = 0.002), diabetes (OR: 2.25, 95% CI 1.21–4.20, P = 0.011), coronary heart disease (OR: 2.91, 95% CI 1.29–6.54, P = 0.010), peripheral vascular disease (OR: 2.86, 95% CI 1.02–7.99, P = 0.045), and the serum levels of Ca × P product (OR: 1.09, 95% CI 1.05–1.13, P < 0.001) and intact PTH (OR: 1.18, 95% CI 1.08–1.28, P < 0.001) were also independently associated with at least one valve calcification.

**Table 4:** Univariate and multivariable analyses for variables associated with at least one valve calcification in the study population (n = 513).

	Univariable		Multivariable	
	Odds ratio (95% CI)	P	Odds ratio (95% CI)	P
Age (1 year)	1.08 (1.06–1.11)	< 0.001	1.05 (1.01–1.09)	0.002
Sex, male	0.88 (0.59–1.31)	0.525	0.77 (0.42–1.39)	0.385
Current smoking	1.07 (0.63–1.83)	0.792		
Diabetes	2.24 (1.47–3.40)	< 0.001	2.25 (1.21–4.20)	0.011
**Cardiovascular disease**				
Coronary heart disease^a^	3.12 (1.99–4.88)	< 0.001	2.91 (1.29–6.54)	0.010
Cerebrovascular disease^b^	2.12 (1.18–3.82)	0.012	1.85 (0.69–4.97)	0.225
Peripheral vascular disease	1.87 (0.94–3.73)	0.077	2.86 (1.02–7.99)	0.045
**Medication**				
ACEI or ARB	1.37 (0.84–2.25)	0.212		
Calcium channel blockers	1.03 (0.69–1.56)	0.873		
Beta-blockers	1.23 (0.82–1.85)	0.320		
Diuretics (thiazide)	0.66 (0.42–1.05)	0.079		
Diuretics (loop)	1.38 (0.92–2.06)	0.120		
Anti-platelet agents	1.21 (0.79–1.85)	0.390		
Statins	1.34 (0.89–2.02)	0.168		
Body mass index (1 kg/m^2^)	1.05 (0.96–1.13)	0.288		
Systolic blood pressure (10 mmHg)	1.53 (1.34–1.75)	< 0.001	1.12 (0.91–1.38)	0.294
Diastolic blood pressure (10 mmHg)	1.47 (1.26–1.71)	< 0.001	1.17 (0.90–1.54)	0.242
eGFR (1 ml/min/1.73 m^2^)	0.93 (0.91–0.95)	< 0.001	1.02 (0.98–1.06)	0.328
Urinary albumin (100 mg/g Cr)	1.03 (1.01–1.04)	0.002	0.99 (0.96–1.02)	0.495
Albumin (1 ng/dl)	0.52 (0.31–0.87)	0.012	1.97 (0.72–5.39)	0.189
Uric acid (1 mg/dl)	1.04 (0.96–1.11)	0.346		
Ca × P product (1 mg^2^/dl^2^)	1.15 (1.11–1.18)	< 0.001	1.09 (1.05–1.13)	< 0.001
Total cholesterol (1 mg/dl)	1.00 (1.00–1.01)	0.694		
Hemoglobin (1 g/dl)	0.72 (0.64–0.82)	< 0.001	1.02 (0.85–1.24)	0.814
CRP (1 mg/dl)	1.80 (1.42–2.28)	< 0.001	1.27 (0.89–1.80)	0.184
Intact PTH (10 pg/ml)	1.22 (1.17–1.27)	< 0.001	1.18 (1.08–1.28)	< 0.001
1,25(OH)_2_D (1 pg/dl)	0.86 (0.84–0.89)	< 0.001	0.88 (0.84–0.92)	< 0.001

Data are presented as odds ratio and 95% confidence interval (CI). ^a^Coronary heart disease is defined as a history of coronary artery bypass surgery or percutaneous transluminal coronary angioplasty. ^b^Cerebrovascular disease is defined as a history of stroke or transient ischemic attack.

*ACEI* angiotensin-converting enzyme inhibitors, *ARB* angiotensin receptor blockers, *Ca × P* product, calcium × phosphorus product, *CKD* chronic kidney disease, *CRP* C-reactive protein, *eGFR* estimated glomerular filtration rate, *PTH* parathyroid hormone, *1,25(OH)*_*2*_*D* 1,25-dihydroxyvitamin D.

### Performance of serum 1,25(OH)_2_D level for predicting the presence of cardiac valve calcification

**Figure 1:**
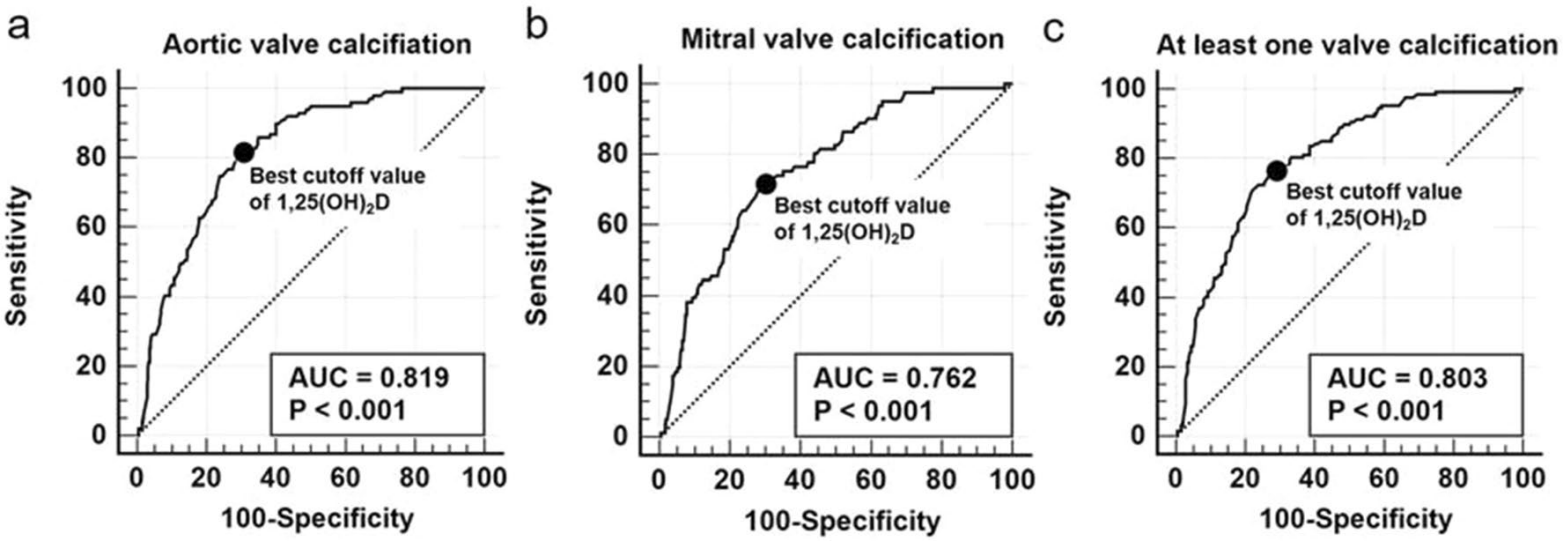
Receiver-operating characteristic curves of serum 1,25(OH)_2_D levels for predicting the presence of AVC (**a**), MVC (**b**) or at least one valve calcification (**c**) in patients with pre-dialysis CKD (n = 513). The areas under the curve for serum 1,25(OH)_2_D levels were 0.819 [95% confidence interval (CI): 0.783–0.852, P < 0.001] for AVC, 0.762 (95% CI 0.722–0.798, P < 0.001) for MVC, and 0.803 (95% CI 0.766–0.837, P < 0.001) for at least one valve calcification. The best cutoff value of serum 1,25(OH)_2_D level for predicting the presence of AVC was ≤ 12.5 pg/dl with an associated sensitivity of 80.8% and specificity of 70.0%. The best cutoff of serum 1,25(OH)_2_D level for predicting the presence of MVC was ≤ 11.9 pg/dl with an associated sensitivity of 71.6% and specificity of 70.8%. Finally, the best cutoff value of serum 1,25(OH)_2_D level for predicting the presence of at least one valve calcification was ≤ 12.5 pg/dl with an associated sensitivity of 76.4% and specificity of 72.3%. AUC, area under the curve; AVC, aortic valve calcification; CI, confidence interval; CKD, chronic kidney disease; MVC, mitral valve calcification; 1,25(OH)_2_D, 1,25-dihydroxyvitamin D.

ROC analysis was performed to investigate the diagnostic power of serum 1,25(OH)_2_D levels in predicting the presence of AVC, MVC, and at least one valve calcification (Fig. 1). The AUCs for serum 1,25(OH)_2_D levels were 0.819 (95% CI 0.783–0.852, P < 0.001) for AVC, 0.762 (95% CI 0.722–0.798, P < 0.001) for MVC, and 0.803 (95% CI 0.766–0.837, P < 0.001) for at least one valve calcification. The best cutoff value of serum 1,25(OH)_2_D level for predicting the presence of AVC was ≤ 12.5 pg/dl with an associated sensitivity of 80.8% and specificity of 70.0%. The best cutoff of serum 1,25(OH)_2_D level for predicting the presence of MVC was ≤ 11.9 pg/dl with an associated sensitivity of 71.6% and specificity of 70.8%. Finally, the best cutoff value of serum 1,25(OH)_2_D level for predicting the presence of at least one valve calcification was ≤ 12.5 pg/dl with an associated sensitivity of 76.4% and specificity of 72.3%.

## Discussion

Vascular calcification has received growing attention in patients with CKD, as accumulating evidence suggests that vascular calcification is one of the major causes of CVD in patients with CKD^18^. However, research has focused on the pathophysiology and clinical impact of vascular calcification as an important part of CKD-MBD, with less attention being paid to cardiac valve calcification in patients with CKD^19^. However, cardiac valve calcification has been reported to be associated with an increased risk of CVD and death in patients with CKD^19^. Therefore, verifying the risk factors for cardiac valve calcification is clinically important for improving the prognosis of patients with CKD. In this study, we investigated the associations between various clinical variables and cardiac valve calcification and found that serum 1,25(OH)_2_D level is an independent risk factor for cardiac valve calcification in patients with CKD.

As the CKD stage increases, cardiac valve calcification is more frequently observed, with the prevalence of AVC increasing from 23% in CKD to 54% in hemodialysis patients, and of MVC, from 25% in CKD to 45% in hemodialysis patients^6,19^. Consistent with previous reports, our study found that the overall prevalence of AVC was 19.3% (8.5%, 25.4%, and 49.2% in patients with CKD stages 3, 4, and 5, respectively) and that of MVC was 15.8% (7.7%, 19.9%, and 39.3% in patients with CKD stages 3, 4, and 5, respectively) in patients with pre-dialysis CKD. The cardiac valve consists of valve endothelial cells and valvular interstitial cells (VICs). Cardiac valve calcification shares common pathophysiological factors with vascular calcification in patients with CKD. Endothelial dysfunction and calcification of interstitial cells of the valve leaflets are the main pathophysiological features of cardiac valve calcification^6^. The process of cardiac valve calcification is complex, and numerous factors can contribute to its pathogenesis and progression. Advanced age, high blood pressure, genetic factors, mechanical stress, metabolic factors (dyslipidemia, diabetes, metabolic syndrome, and metabolic uremic factors), inflammation, mineral/hormone-related factors (hyperphosphatemia and Ca × P product and PTH levels), and drugs, including calcium-based phosphate binders, have been suggested as risk factors for cardiac valve calcification in patients with CKD^6,18–20^. Consistent with the findings of previous studies, the multivariable analyses in our study showed that age (for AVC and MVC), systolic blood pressure (for AVC), coronary heart disease (for AVC and MVC), diabetes (for AVC and MVC), and Ca × P product (for AVC and MVC) and intact PTH levels (for AVC and MVC) were independently associated with cardiac valve calcification in patients with CKD.

Previous studies have suggested the potential role of vitamin D and in various types of kidney disease. Vitamin D insufficiency is common and leads to secondary hyperparathyroidism in patients with CKD. Vitamin D compounds remain the first-line therapy for the treatment of secondary hyperparathyroidism (SHPT)^21^. Vitamin D is known to be associated with renal tubular homeostasis. The protective role of vitamin D against chronic inflammation observed in tubular injury has been demonstrated in animal and human studies^22^. In glomerulonephritis, vitamin D preserves the structural integrity of the slit diaphragm, significantly prevents the loss of nephrin, podocin, and tight junction protein^23^. In diabetic nephropathy, growing evidence has suggested that vitamin D might have anti-proteinuric, anti-inflammatory, and renoprotective effects^24^. Finally, prospective randomized studies are needed to determine the role of vitamin D in those kidney diseases.

The main finding of this study was that a low level of serum 1,25(OH)_2_D is independently associated with the presence of AVC and MVC in patients with CKD. Concerning the association between vitamin D deficiency and cardiac valve calcification, several studies have reported this association in patients with diseases other than CKD. Dishmon et al. showed that low levels of serum 25(OH)D are associated with cardiac valve calcification in patients with dilated cardiomyopathy without significant renal dysfunction^25^. Yusuf et al. reported that serum 25(OH)D levels correlated with the severity of valvular calcification in patients with rheumatic mitral stenosis^26^. Tibuakuu et al. reported a possible link between serum 25(OH)D level and the risk of incident mitral annulus calcification, but not AVC, in the general population free of preexisting clinical CVD^10^. To our knowledge, this is the first study to report an association between serum vitamin D deficiency (measured by serum 1,25(OH)_2_D levels) and cardiac valve calcification in patients with CKD.

Our study demonstrated an independent association between serum 1,25(OH)_2_D and cardiac valve calcification even after adjustment for several confounding factors of CKD-MBD (calcium, phosphate, Ca × P product, and intact PTH levels), suggesting a direct causal role of low serum 1,25(OH)_2_D levels in cardiac valve calcification in patients with CKD. However, the pathophysiological explanation for the association between serum 1,25(OH)_2_D levels and cardiac valve calcification in patients with CKD is still unclear. One possible mechanism is that vitamin D may be involved in the differentiation of VICs into osteoblast-like cells. VICs are a major cellular component of cardiac valve leaflets. As valvular calcification progresses, a subpopulation of VICs undergoes a phenotypic transformation into osteoblast-like cells^27^. Osteoblast-like cells may be involved in the development of valvular calcification as osteoblasts play a central role in bone development^27^. Schmidt et al. showed that a low vitamin diet accelerated valvular calcification by differentiating VICs into osteoblast-like cells in an animal model, suggesting a causal role of vitamin D deficiency in valvular calcification^28^. In another report by Schmidt et al., vitamin D receptor (VDR) deficiency promoted AVC in a VDR^−/−^ mouse model via upregulation of osteoblast transcription factors, which triggered the differentiation of VICs into osteoblast-like cells^28^. Another potential mechanism underlying the vitamin D–cardiac valve calcification association is inflammation. It is well established that inflammation promotes valvular calcification^6,19^. VDR is abundantly expressed in immune cells, and vitamin D has potent anti-inflammatory properties^29^. While physiological levels of vitamin D are capable of inhibiting calcification by modulating inflammation, vitamin D deficiency observed in patients with CKD leads to a pro-inflammatory activity that may subsequently drive calcification^30^.

However, the question remains whether vitamin D supplements can delay the progression of cardiac valve calcification in patients with CKD. With regard to vascular calcification, the 2017 KDIGO guidelines on CKD-MBD recommend avoiding calcitriol and vitamin D analog supplementation in patients with CKD not on dialysis because excessive vitamin D supplementation can cause hypercalcemia and hyperphosphatemia, which may promote vascular calcification^31^. If initiated for severe and progressive SHPT, calcitriol or vitamin D analogs should be started with low doses, and then titrated based on the PHT response. Thus, the 2017 KDIGO guidelines suggest that calcitriol and vitamin D analogs not be routinely used in patients with CKD not on dialysis^31^. As to cardiac valve calcification, excessive vitamin D supplementation was shown to be associated with AVC in an animal model^32^. Further clinical studies are needed to verify the effect of vitamin D supplementation on cardiac valve calcification in patients with CKD.

There were several limitations to the present study. First, owing to its retrospective and cross-sectional design, it was difficult to establish a temporal and causal relationship between serum 1,25(OH)_2_D levels and cardiac valve calcification. Further experimental and clinical studies are needed to establish the causal relationship between serum 1,25(OH)_2_D levels and cardiac valve calcification in patients with CKD. Second, our study was performed at a single center and had a relatively small sample size. Thus, our results may not be extrapolated to the overall population with CKD. Third, this study did not include fibroblast growth factor (FGF)23, which is one of the major components of CKD-MBD, in the analysis due to the retrospective design of this study. FGF23 is known to inhibit 1α-hydroxylase. Early in CKD, the decline in 1,25(OH)_2_D levels is likely due to the increase in FGF23 levels rather than the loss of functional renal mass^33^. Thus, if FGF23 levels had been included in the analysis, the association between serum 1,25(OH)_2_D and cardiac valve calcification could have been addressed in greater detail.

Nevertheless, the present study had several strengths. First, serum 1,25(OH)_2_D levels were measured instead of those of 25(OH)D. The levels of 1,25(OH)_2_D reflect the true biological activity of vitamin D because this form binds to VDR, whereas 25(OH)D levels reflect the vitamin D stores because it is the main circulating form of vitamin D^34^. We think that the results of this study are more physiologically relevant than those of previous studies that measured 25(OH)D levels. Second, to unveil the association between vitamin D and cardiac valve calcification more clearly, we excluded the patients who were taking vitamin D supplements, calcimimetics, and phosphate binders, which could affect endogenous vitamin D metabolism. Third, we showed not only an independent association between serum 1,25(OH)_2_D levels and cardiac valve calcification but also the best cutoff values of serum 1,25(OH)_2_D levels to predict the presence of cardiac valve calcification, suggesting that serum 1,25(OH)_2_D levels may be a biomarker for cardiac valve calcification in patients with CKD.

In conclusion, our study demonstrates that serum 1,25(OH)_2_D level is independently associated with cardiac valve calcification and may be a potential biomarker for cardiac valve calcification in patients with CKD. Future studies are needed to demonstrate the role of vitamin D in the pathogenesis of cardiac valve calcification in CKD and to determine whether vitamin D therapy can prevent the progression of cardiac valve calcification in patients with CKD.

## Supplementary Information

Supplementary Information.

## Data Availability

All data generated or analyzed during this study are included in this published article and its Supplementary Information files.
