# Supplementary material for: 1,25-dihydroxyvitamin D deficiency is independently associated with cardiac valve calcification in patients with chronic kidney disease
Source: Sci Rep. 2022 Jan 18;12:915. doi: 10.1038/s41598-022-04981-x (PMC8766529; doi:10.1038/s41598-022-04981-x)
Supplement: Supplementary file 1 — Supplementary Information. [file 41598_2022_4981_MOESM1_ESM.docx]

**1,25-dihydroxyvitamin D deficiency is independently associated with cardiac valve calcification in patients with chronic kidney disease**

Il Young Kim^1,2^, Byung Min Ye^1,2^, Min Jeong Kim^1,2^, Seo Rin Kim^1,2^, Dong Won Lee^1,2^, Hyo Jin Kim^1,3^, Harin Rhee^1,3^, Sang Heon Song^1,3^, Eun Young Seong^1,3^, and Soo Bong Lee^1,2*^

^1^Department of Internal Medicine, Pusan National University School of Medicine, Yangsan, South Korea ^2^Research Institute for Convergence of Biomedical Science and Technology, Pusan National University Yangsan Hospital, Yangsan, South Korea

^3^Medical Research Institute, Pusan National University Hospital, Busan, South Korea

^*^Correspondence: E-mail: sbleemd@pusan.ac.kr

**Supplement Table S1**. Baseline characteristics of study population according to CKD stage (n = 513).

|  | CKD stage 3  (n = 271) | CKD stage 4  (n = 181) | CKD stage 5  (n = 61) | P |
| --- | --- | --- | --- | --- |
| Age (years) | 57.9 ± 9.3 | 62.8 ± 9.99 | 64.6 ± 11.5 | <0.001 |
| Sex, male [n (%)] | 138 (50.9%) | 94 (51.9%) | 31 (50.8%) | 0.975 |
| Current smoking [n (%)] | 52 (19.2%) | 25 (13.8%) | 8 (13.1%) | 0.239 |
| Diabetes [n (%)] | 136 (50.2%) | 94 (51.9%) | 34 (55.7%) | 0.726 |
| Cardiovascular disease [n (%)] |  |  |  |  |
| Coronary heart disease^a^ | 43 (15.9%) | 45 (24.9%) | 23 (37.7%) | <0.001 |
| Cerebrovascular disease^b^ | 21 (7.7%) | 21 (11.6%) | 12 (19.7%) | 0.020 |
| Peripheral vascular disease | 13 (4.8%) | 17 (9.4%) | 8 (13.1%) | 0.036 |
| Medication [n (%)] |  |  |  |  |
| ACEI or ARB | 199 (73.4%) | 140 (77.3%) | 52 (85.2%) | 0.133 |
| Calcium channel blockers | 151 (55.7%) | 117 (64.6%) | 44 (72.1%) | 0.025 |
| Beta-blockers | 85 (31.4%) | 78 (43.1%) | 32 (52.5%) | 0.002 |
| Diuretics (thiazide) | 108 (39.9%) | 38 (21.0%) | 7 (11.5%) | <0.001 |
| Diuretics (loop) | 100 (36.9%) | 84 (46.4%) | 36 (59.0%) | 0.003 |
| Anti-platelet agents | 75 (27.7%) | 60 (33.1%) | 23 (37.7%) | 0.073 |
| Statins | 95 (35.1%) | 67 (37.0%) | 24 (39.3%) | 0.501 |
| Body mass index (kg/m^2^) | 23.7 ± 2.5 | 23.7 ± 2.5 | 23.4 ± 2.1 | 0.624 |
| Systolic blood pressure (mmHg) | 129.6 ± 19.8 | 138.5 ± 18.8 | 143.8 ± 14.3 | <0.001 |
| Diastolic blood pressure (mmHg) | 77.1 ± 13.0 | 81.2 ± 14.8 | 82.8 ± 14.0 | 0.001 |
| eGFR (ml/min/1.73 m^2^) | 42.2 ± 8.2 | 22.1 ± 4.6 | 10.2 ± 4.5 | <0.001 |
| Urinary albumin (mg/g Cr) | 835.0 ± 807.4 | 1429.9 ± 1141.3 | 2458.0 ± 1489.9 | <0.001 |
| Albumin (g/dl) | 4.2 ± 0.3 | 4.0 ± 0.4 | 3.8 ± 0.5 | <0.001 |
| Uric acid (mg/dl) | 6.7 ± 2.5 | 7.8 ± 2.9 | 9.0 ± 2.0 | <0.001 |
| Calcium (mg/dl) | 9.3 ± 0.4 | 9.2 ± 0.5 | 9.0 ± 1.1 | <0.001 |
| Phosphate (mg/dl) | 3.7 ± 0.8 | 4.3 ± 0.8 | 5.4 ± 0.9 | <0.001 |
| Ca × P product (mg^2^/dl^2^) | 34.5 ± 7.4 | 39.5 ± 8.4 | 48.0 ± 9.7 | <0.001 |
| Total cholesterol (mg/dl) | 209.5 ± 41.7 | 212.7 ± 42.2 | 214.0 ± 39.2 | 0.627 |
| Hemoglobin (g/dl) | 12.7 ± 1.5 | 11.1 ± 1.6 | 9.6 ± 1.0 | <0.001 |
| CRP (mg/dl) | 0.6 ± 0.8 | 0.8 ± 0.7 | 1.4 ± 1.1 | <0.001 |
| Intact PTH (pg/ml) | 52.7 ± 32.3 | 110.1 ± 40.3 | 198.2 ± 59.6 | <0.001 |
| 1,25(OH)_2_D (pg/dl) | 22.0 ± 10.7 | 15.1 ± 9.1 | 8.3 ± 3.9 | <0.001 |
| Aortic valve calcification [n (%)] | 23 (8.5%) | 46 (25.4%) | 30 (49.2%) | <0.001 |
| Mitral valve calcification [n (%)] | 21 (7.7%) | 36 (19.9%) | 24 (39.3%) | <0.001 |
| At least one valve calcification [n (%)] | 35 (12.9%) | 58 (32.0%) | 34 (55.7%) | <0.001 |

Data are presented as mean ± standard deviation or (n, %). ^a^Coronary heart disease is defined as a history of coronary artery bypass surgery or percutaneous transluminal coronary angioplasty. ^b^Cerebrovascular disease is defined as a history of stroke or transient ischemic attack. ACEI, angiotensin-converting enzyme inhibitors; ARB, angiotensin receptor blockers; Ca × P product, calcium × phosphorus product; CKD, chronic kidney disease; CRP, C-reactive protein; eGFR, estimated glomerular filtration rate; PTH, parathyroid hormone; 1,25(OH)_2_D, 1,25-dihydroxyvitamin D
